# The Persistence of Hepatitis C Virus Infection in Hepatocytes Promotes Hepatocellular Carcinoma Progression by Pro-Inflammatory Interluekin-8 Expression

**DOI:** 10.3390/biomedicines9101446

**Published:** 2021-10-11

**Authors:** Ciniso Sylvester Shabangu, Phumelele Yvonne Siphepho, Chia-Yang Li, Wei-Chung Cheng, Ming-Ying Lu, Chung-Feng Huang, Ming-Lun Yeh, Chia-Yen Dai, Jee-Fu Huang, Wan-Long Chuang, Zu-Yau Lin, Ming-Lung Yu, Shu-Chi Wang

**Affiliations:** 1 Graduate Institute of Medicine, Kaohsiung Medical University, Kaohsiung 80708, Taiwan; u107567006@kmu.edu.tw (C.S.S.); chiayangli@kmu.edu.tw (C.-Y.L.); beechy@ms57.hinet.net (M.-Y.L.); 2 Center for Cancer Research, Center for Liquid Biopsy and Cohort Research, Kaohsiung Medical University, Kaohsiung 80708, Taiwan; jfliver@kmu.edu.tw (J.-F.H.); fish6069@gmail.com (M.-L.Y.); 3 Program in Tropical Medicine, Graduate Institute of Medicine, Kaohsiung Medical University, Kaohsiung 80708, Taiwan; yolliesiphepho@gmail.com; 4 Research Center for Cancer Biology, Graduate Institute of Biomedical Science, China Medical University, Taichung 406040, Taiwan; cwc0702@gmail.com; 5 Hepatobiliary Division, Department of Internal Medicine, Kaohsiung Medical University Hospital, Kaohsiung Medical University, Kaohsiung 80708, Taiwan; fengcheerup@gmail.com.tw (C.-F.H.); minglunyeh@gmail.com (M.-L.Y.); daichiayen@gmail.com (C.-Y.D.); waloch@kmu.edu.tw (W.-L.C.); zuyali@cc.kmu.edu.tw (Z.-Y.L.); 6 Faculty of Internal Medicine, School of Medicine, College of Medicine, Kaohsiung Medical University, Kaohsiung 80708, Taiwan; 7 Hepatitis Research Center, Kaohsiung Medical University, Kaohsiung 80708, Taiwan; 8 Department of Medical Laboratory Science and Biotechnology, Kaohsiung Medical University, Kaohsiung 80708, Taiwan; 9 Department of Medical Research, Kaohsiung Medical University Hospital, Kaohsiung 80756, Taiwan

**Keywords:** hepatitis C virus (HCV), hepatocellular carcinoma (HCC), C-X-C motif ligand 8 (CXCL8), proto-oncogene tyrosine-protein kinase Src (SRC)

## Abstract

Background: A large amount of epidemiological evidence indicates that persistent HCV infection is the main risk factor for HCC. We aimed to study the effects of persistent HCV infection on the interaction of the virus and host cell to identify cancer gene profiles. Methods: Next-generation sequencing (NGS) was used to identify differentially expressed genes between uninfected Huh7.5.1 control cells, short-term HCV (S-HCV), early long-term HCV (eL-HCV), and long-term HCV (L-HCV) infections, which were analyzed using different dynamic bioinformatics and analytic tools. mRNA expression was validated and quantified using q-PCR. One hundred ninety-six serum samples of HCV patients with IFN/RBV treatment were used to study chemokine levels. Results: S-HCV activates an inflammatory response and drives cell death and apoptosis through cell cycle arrest via MAPK signaling. L-HCV promotes cell growth and alters cell adhesion and chemokine signaling via CXCL8-mediated-SRC regulation. A total of 196 serum samples from the HCV and HCV-HCC cohorts demonstrated significantly upregulated pro-inflammatory CXCL8 in non-SVR (persistent HCV infection) patients in the HCV-HCC group. Conclusions: Persistent infection with HCV induced pro-inflammatory CXCL8 and the oncogene SRC, thereby triggering and promoting hepatocarcinogenesis. CXCL8 may be a potential biomarker for monitoring HCV-related HCC progression.

## 1. Introduction

Hepatitis C virus (HCV)-associated mortality within the HCV population is estimated to be 6.3%, which is 4.7 times higher compared to the general population [1]. The diagnoses of an HCV infection is assessed based on virologic criteria from laboratory serological assays to detect specific HCV antibodies (indirect tests) and through the detection or quantification of components of HCV viral particles (direct tests) [2]. According to the WHO, approximately 30 percent (15–45%) of infected people spontaneously clear the HCV virus in less than 6 months of infection with no treatment. The outstanding 70 percent (55–85%) of patients progress to chronic HCV infection (CHC), with an increasing risk of cirrhosis, differing from 15% to 30% within 20 years [3]. Even with the early diagnosis of HCV infection, the early treatment of patients with chronic hepatitis would decrease the total number of CHC cases by an estimated 7% to 11% [4]. Even with the consideration of this decrease in CHC cases, this still fails to account for undiagnosed or untreated HCV patients. The long-term persistence of HCV confers resistance to treatment regimens such as the NS5A inhibitors ledipasvir and daclatasvir, in which HCV infection persisted at detectable levels for >96 weeks in patients who failed to achieve SVR [5]. Furthermore, as a limitation of the current testing methods, HCV persisted at levels that are undetectable in patients who had spontaneous HCV clearance, suggesting that the host’s immune response on its own cannot eliminate the virus [6]. Since HCV induces epigenetic changes which are risk factors for HCC, it is imperative to understand the dynamic relationship between HCV and HCC in patients [7].

HCV, a lipid-centric virus, regulates viral ingress into permissible host cells through interactions with surface proteins and receptors [8]. In the cytoplasm, the HCV genome is exposed to the host’s immune mechanisms and translates large polyproteins which are broken down by cellular and viral proteases into structural and nonstructural proteins in endoplasmic reticulum (ER)-related modifications [9]. A previous study indicated that host factors assist in HCV replication to increase the efficiency of translation and interactions with viral NS5A and NS5B to increase HCV replication [10]. These may correlate to the induction of specific genome-wide changes which promote the risk of liver cancer [11]. A study demonstrated how HCV (JFH1) derived from a viral isolate of a patient with fulminant hepatitis C replicated efficiently in human hepatoma (Huh7) cells without adaptive mutations [12]. Furthermore, research shows that the transfection of full length JFH1 genomes into Huh7 or Huh7-derived cells leads to cell secretion of the infectious virus. Successful infection was demonstrated via the detection of viral proteins and a highly reproducible time-based increase in viral RNA in infectious cells [13,14]. Viral core positive cells reflect the extent of HCV-infected cells. About 80% of the cells post-infection indicated a high rate of HCV replication, corresponding to the early/acute phase of infection, whereas in the late/chronic stage of infection (28 to 76 days post-infection) only about 15% of the cells expressed the HCV core protein [15]. HCV RNA in the intracellular compartment reached the highest titer after 6–8 days post-infection and gradually decreased during the chronic phase, which was consistent with a persistently infected cell culture. Furthermore, apoptotic death in infected cell cultures has been linked to a high intracellular viral RNA titer, suggesting a causative interaction between the rate of HCV replication and cell death. However, a limited number of studies have focused on the long-term viral infection in infected cells and the relationship between HCV and HCC. The aim of this study was to investigate the long-term and short-term effects of HCV-infection-induced gene regulation and expression on the interaction between the virus and host cells. Through dynamic analysis tools, we determined the effects of short-term HCV (S-HCV), early long-term HCV (eL-HCV), and long-term HCV (L-HCV) on host cell gene expression to alter liver microenvironments. A flowchart of our study design is illustrated in Figure S1.

## 2. Materials and Methods

### 2.1. Cell Culture and HCV Viral Infection

The human hepatoma Huh7.5.1 cell line was cultured and incubated in a humidified 5% CO_2_/95% air atmosphere at 37 °C to reach 5 × 10^6^ cells in a 10 cm Petri dish with Dulbecco’s modified Eagle medium (DMEM) (Cat. # 21969035 Thermo Fisher Scientific, Waltham, MA, USA) mixed with 10% fetal bovine serum (FBS) (Lonza, Walkersville, MD, USA), 2% L-glutamate containing 200 mM solution in 0.85% NaCl solution (Lonza, Walkersville, MD, USA) and 2% antibiotics containing 10,000 U Pen./mL, 10,000 ug Strep./mL, 25 ug Amphotericin B/mL (Lonza, Walkersville, MD, USA) and 1% Non-Essential Amino Acids (NEAA) (Lonza, Walkersville, MD, USA). In the range of 1–10 passage numbers, 5 × 10^5^ of the Huh7.5.1 cells in the 10 cm Petri dish were infected via incubation with HCV JFH1-EYFP virus with a 1:200 ratio (viral medium: fresh medium) (HCV cc, developed by Dr. Machi Yamamoto) for 6 h, after which the medium was replaced with fresh medium and cells were incubated for 5 days.

### 2.2. Distinguishing Intracellular HCV via Fluorescence-Activated Cell Sorting (FACS)

To establish the different HCV infectious cell populations, we modified a previously published model that used fluorescence-activated cell sorting (FACS) in which HCV infectious cells were distinguished into high and low viral loads by the amount of virus within the cells [16]. The *x*-axis (counts) represents the number of cells presenting a viral presence, whereas the *y*-axis (FITC-Log_Height) represents the amount of JFH-EYFP viral fluorescence (Figure S2A). In this study, HCV JFH1-EYFP infectious cells were distinguishable into two cell populations, those with low viral loads (R1 region, 28% of infectious cells) and those with high viral loads (R2 region, 26% of infectious cells), based on their fluorescence intensity after 5 days of infection (Figure S2A).

The infectious cells were gated at Log 10^0^–Log 10^1.5^ of FITC intensity for low viral intensity to obtain low-viral-load cells and Log 10^2.5^–Log 10^5^ of FITC intensity for high viral intensity to obtain high-viral-load cells by means of a dot plot (Figure S2B). After FACS, we rechecked and confirmed the distinct populations, which showed an FITC mean fluorescence intensity (MFI) of 78.4 for the total isolated low-viral-load cells and 826 for the total isolated high-viral-load cells, respectively (Figure S2C,D). Low-viral-load cells, from here on referred to as “early long-term HCV (eL-HCV)” and high-viral-load cells, from here on referred to as “short-term HCV (S-HCV)”, were confirmed for intracellular EYFP intensity by means of fluorescence microscopy at 100× *g* after cell sorting (Figure S2E,F). S-HCV presented high viral fluorescence, whereas eL-HCV presented low viral fluorescence, demonstrating the efficiency of viral replication in cells and also the relationship between viral load and viral replication. S-HCV and eL-HCV represented the short-term effects of HCV infection, which is associated with the early phase of HCV infection. To establish long-term infectious cells which would represent persistent infection, eL-HCV cells from day 5 were cultured to day 30 and showed 23.5% high-viral-load (R2 region) cells (Figure S3A,B). We sorted the R4 region of low-viral-load cells and confirmed them to be a distinct population of long-term-HCV (L-HCV) cells (R4 region), referred to as long-term HCV (L-HCV), based on FACS. (Figure S3C).

### 2.3. Next-Generation Sequencing (NGS)

In preparation for NGS, the control group of uninfected-Huh751 cells and three sample groups (S-HCV, eL-HCV, L-HCV) were extracted for RNA. Total RNA was extracted from cells using TRIzol reagent (Thermo Fisher Scientific, Waltham, MA, USA) following the manufacturer’s recommendations. The quality of the total RNA was evaluated using an Agilent 2100 bioanalyzer (Agilent, Palo Alto, CA, USA) using an RNA 6000 Nano LabChip kit (Agilent, Palo Alto, CA, USA). To construct the lncRNA libraries, ribosomal RNA was depleted using a Ribo-Zero Gold rRNA Removal Kit (cat. no.: MRZG126: Illumina, San Diego, CA, USA). First-strand cDNA was prepared using the SMARTer Stranded RNA-Seq Kit (cat. no.: 634836, Takara Bio, Shiga-ken, Japan) and SMART stranded N6 primer. First-strand templates were reverse-transcribed into double-stranded cDNA. To prepare indexed samples for NGS, cDNA was PCR-amplified using an Illumina index primer set (Illumina, San Diego, CA, USA). The indexed libraries were cleaned using Agencourt AMPure XP beads (cat. no. A63881, Beckman Coulter Genomics, Chaska, MN, USA), and their sizes were determined using an Agilent 2100 bioanalyzer (cat. no. G2943CA, Agilent, Palo Alto, CA, USA) and a DNA 1000 Kit (cat. no. 5067-1504, Agilent, Palo Alto, CA, USA). Pooled libraries were subjected to high-throughput 125-bp paired-end sequencing on an Illumina HiSeq system (Illumina, San Diego, CA, USA). The amplified RNAs, following the construction of a cDNA library, were subjected to RNA-Seq on an Illumina Genome Analyzer II (Illumina, San Diego, CA, USA) for paired-end sequencing. Upon completion, approximately 14.9–20.5 × 10^6^ of 75-bp-long sequence reads per sample were generated, corresponding to an average of 1.30 Gb raw sequence data. The reads were aligned to GRCh37 in TopHat 2.0.13, with default parameters, using Ensembl v75 annotations. Transcript abundance was measured in fragments per kilobase of exon per million mapped fragments. After NGS, the expression of the identified genes in each sample were compared to the control group for relative expression as illustrated below:

### 2.4. Relative Fold Change Expression

Relative Expression (∆∆Ct) = (Sample ∆Ct)/(Control ∆Ct)(1)

Relative Expression Fold Change (FC) = 2^−(∆∆*C*t)^ (2)

### 2.5. Data Analysis Tools

Our candidate genes were analyzed using different bioinformatics methods for an in-depth understanding of their roles. Ingenuity Pathway Analysis (IPA) software analysis results were used to identify our gene lists’ associated network functions. The identified associated network functions and genes were further analyzed by GenCLiP 3, a web server version analysis tool; from this analysis we identified functional gene clusters [17]. The top enriched functional clusters of genes were analyzed using ShinyGO, an intuitive and graphical web application, to identify the top enriched KEGG results [18]. The Search Tool for the Retrieval of Interacting Genes (STRING) database (version 11) contains analysis and combines protein–protein interactions (PPI) [19]. Gene Expression Profiling Interactive Analysis (GEPIA), a customizable and interactive tool, was used for differential expression analysis and patient survival analysis [20]. For more details about the analytical tools used, refer to the Supplementary Materials.

### 2.6. Western Blotting

Total protein was extracted using RIPA protein lysis buffer (cat. no. 89900, Thermo Fisher, USA) with freshly added 1% protease inhibitor cocktail and 1 mM phenylmethylsulfonyl fluoride (PMSF) for 30 min on ice and then centrifuged at 13,000× *g* for 20 min. A total of 10 μg of protein was determined using the Bio-Rad protein assay and used for Western blotting. After Western blotting, the samples were separated using SDS-PAGE and transferred to PVDF membranes. PVDF membrane blots were blocked with 5% skim milk, followed by incubation with primary antibodies for HCV NS3 (MAB8691, Merck Millipore, KGaA, Germany), SRC (AHO0051, Thermo Fisher, USA), and actin (clone AC-40, A3853, Sigma-Aldrich) overnight at 4 °C in blocking buffer. Finally, the membrane blots were conjugated with horseradish peroxidase (HRP) secondary antibodies for 2 h and the signals were developed with chemiluminescence reagents (Amersham Biosciences, CA, USA). Chemiluminescence-associated bands were identified using ECL (Bio-Rad, ChemiDoc XRS + System) and quantified.

### 2.7. Cohort Collection

Chronic hepatitis C patients receiving antiviral treatment from Kaohsiung Medical University Chung-Ho Memorial Hospital (KMUH) were consecutively recruited in a prospective follow-up cohort between 2002 and 2012 of 196 patients. Peginterferon alpha-2a/alpha-2b plus ribavirin (IFN/RBV) was administered to all the recruited participants. HIV or hepatitis B virus infection co-infected patients were excluded, as were patients who showed alcohol abuse (≥20 g daily) and patients who exhibited signs of HCC pre-antiviral, during or within 6 months post-antiviral treatment. Sera negative for HCV RNA were used to define SVR or without-SVR patients during a 24-week follow-up post-antiviral treatment. The patients were further assessed for the prospect of HCC development. Liver biopsies were performed less than 6 months before starting antiviral treatment to evaluate cirrhosis, and the Knodell and Scheuer scoring system was used to grade and stage liver histology [21]. Level of cirrhosis and treatment results were used as a follow-up approach after treatment, as previously described [22]. Follow-up was every 3 months if patients were cirrhotic/non-SVR and every 6–12 months if patients were non-cirrhotic and had achieved SVR. HCC was confirmed by either histology or imaging, combined with laboratory evidence, based on the Asian Pacific Association for the Study of the Liver [23] and the American Association for the Study of Liver Diseases guidelines [24].

### 2.8. Real-Time PCR

Total RNA was isolated using TRIzol reagent. cDNA was synthesized from 1 μg of RNA using a High-Capacity cDNA Reverse Transcription kit (Applied Biosystems, Foster City, CA, USA). Real-time PCRs were run in triplicate for each sample in a single 20 µL reaction using Power SYBR Green (Thermo Fisher, Waltham, MA, USA) in an ABI PRISM 7900 fast detection system (Thermo Fisher, Waltham, MA, USA). Target gene expression was normalized to that of the house-keeping gene *GAPDH*, calculated via the threshold cycle (Ct) method using the ABI 7900 system software (Thermo Fisher, Waltham, MA, USA). The primers of the genes are presented in Table S1.

Fold change expression was calculated using the equation:

∆Ct (normalization) = (gene of interest(GOI)) Ct − (housekeeping gene) Ct(3)

∆∆Ct = (normalized GOI in Sample) ∆Ct − (normalized gene in Control) ∆Ct(4)

Fold gene expression = 2^−(∆∆*C*t)^ (5)

### 2.9. Chemokine Assays

Sera from 196 patients with HCV-HCC and HCV-non-HCC in the cohort treated with pegylated interferon (IFN) from Kaohsiung Medical University Chung-Ho Memorial Hospital (KMUH) were centrifuged at 300× *g* for 15 min and stored at −80 °C. CXCL8 levels were quantified using ELISA (eBioscience, MA, USA) via a 1:50 sample dilution. The variables were compared between groups using the Wilcoxon test, using JMP14.

### 2.10. Statistical Analysis

Statistical analyses were performed using a two-tailed unpaired *t*-test to determine statistical significance. *p*-values < 0.05 were deemed statistically significant. Calculations were performed using GraphPad Prism 8. For HCV-HCC patients in the cohort treated with pegylated interferon (IFN), classifiable variables were compared between groups using the Wilcoxon test, using JMP14.

## 3. Results

### 3.1. S-HCV Is Implicated in Cell Death and Survival Network, Whereas L-HCV Is Implicated in Cellular Movement, Cell-To-Cell Signalling, and the Cancer Network

**Figure 1:**
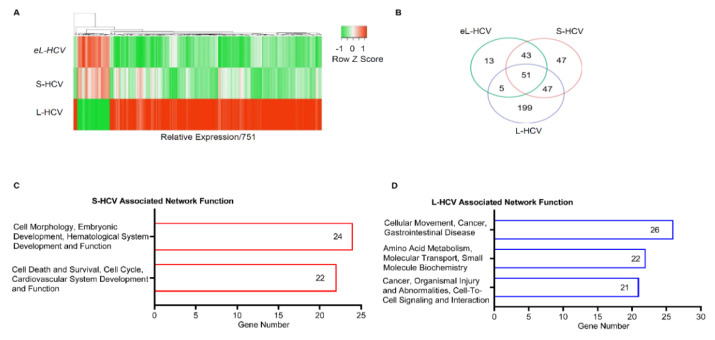
Intracellular HCV altered gene expression and associated diseases and bio-functions. (**A**) The heatmap demonstrating altered gene expression relative to uninfected Huh7.5.1 cells, comparing short-term HCV (S-HCV), early long-term (eL-HCV), and long-term HCV (L-HCV) using NGS gene expression data. (**B**) A Venn diagram showing the number of genes associated with S-HCV, eL-HCV, and L-HCV after a cutoff fold change ≥−2 or ≤2, *p* < 0.05, based on NGS expression data. (**C**) Ingenuity pathway analysis (IPA) results of associated network functions showing the number of viral-load-exclusive genes implicated in the networks of S-HCV (genes = 47) and (**D**) the networks of L-HCV (genes = 199).

To study the regulation of genes in the presence of intracellular HCV, the differentially expressed mRNA in S-HCV, eL-HCV, and L-HCV were analyzed. Uninfected Huh7.5.1 cells were used as a control to calculate the relative expression of S-HCV, eL-HCV, and L-HCV, and the results were presented as a heatmap. The heatmap showed a similar expression pattern between S-HCV and eL-HCV, whereas L-HCV demonstrated a completely different gene expression pattern when compared to both S-HCV and eL-HCV (Figure 1A). These results suggest that HCV infection duration and viral loads regulate gene expression, notably in L-HCVs. Subsequent analysis showed that 47, 13, and 199 genes, (fold change ≥ −2 or ≤ 2), (*p* < 0.05) were significantly differentially expressed in S-HCV, eL-HCV, and L-HCV, respectively, as illustrated in the Venn diagram (Figure 1B and Table S2). Intersecting genes (S-HCV/eL-HCV/L-HCV = 51 genes, S-HCV/eL-HCV = 43 genes, eL-HCV/L-HCV = 5 genes, S-HCV/L-HCV = 47 genes) were excluded from further analysis. Thirteen eL-HCV-exclusive genes demonstrated a similar expression profile to S-HCV, and were excluded from further analysis. We therefore focused on S-HCV- and L-HCV-exclusive genes for in-depth analysis to understand the mechanisms and functions linked with short-term HCV infection and long-term HCV infection, respectively. Analysis of the forty-seven S-HCV-exclusive genes revealed that they were associated with “Cell Morphology, Embryonic Development, Hematological System Development and Function” (genes = 24/47, 51.06%, score = 47) and “Cell Death and Survival, Cell Cycle, Cardiovascular System Development and Function” (genes = 22/47, 46.81%, score = 42) (Figure 1C and Table 1). One hundred ninety-nine of the L-HCV-exclusive genes were associated with “Cellular Movement, Cancer, Gastrointestinal Disease” (gene = 26/199, 13.07%, score = 43); Amino Acid Metabolism, Molecular Transport, Small Molecule Biochemistry (gene = 22/199, 11.06%, score = 34) and “Cancer, Organismal Injury and Abnormalities, Cell-to-Cell Signaling and Interaction” (gene = 21/199, 10.56%, score= 32) (Figure 1D and Table 2). These results suggest that the most differentially expressed genes in S-HCV were involved in cell death and survival networks, whereas L-HCV genes were involved in cellular movement, cancer, and gastrointestinal disease networks.

### 3.2. S-HCV Is Driven towards Cell Death and an Inflammatory Response

**Figure 2:**
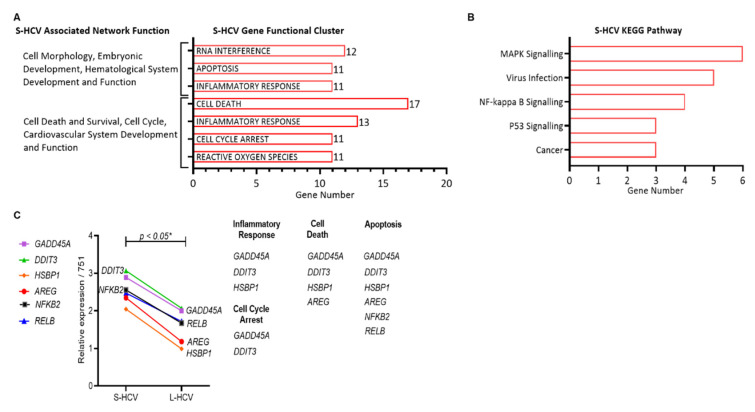
Short-term HCV (S-HCV)-associated network function gene analysis. (**A**) Short-term HCV (S-HCV)-associated network functions and GenClip gene functional clusters (*p* < 0.05). (**B**) ShinyGO Kyoto Encyclopedia of Genes and Genomes (KEGG) analysis of the top 6 enriched pathways (*p*-value cutoff (FDR) = 0.05) ranked by number of genes from highest to lowest. (**C**) Expression of five genes (*GADD45A*, *DDIT3*, *HSBP1*, *AREG*, *NFKB2*) in the mitogen-activated protein kinase (MAPK) signaling pathway compared between S-HCV and L-HCV, statistical significance *p* < 0.05 *. MAPK signaling genes grouped by the functional clusters.

To determine the mechanisms by which S-HCV was directed towards cell death and survival, we identified the top enriched gene functional clusters of S-HCV-associated functions network genes, as shown in Table 1. The significantly enriched clusters (enrichment scores (ESs) with *p* < 0.05) were ranked based on the highest number of genes per associated network function. Twenty-four genes associated with “Cell Morphology, Embryonic Development, Hematological System Development and Function” were clustered to “RNA interference” (genes = 12/24, ES = 9.35, *p* = 2.217 × 10^−9^), “apoptosis” (genes = 11/24, ES = 10.99, *p* = 1.854 × 10^−11^), and “inflammatory response” (genes = 11/24, ES = 9.70, *p* = 7.637 × 10^−10^) (Figure 2A). Twenty-two genes associated with the “Cell Death and Survival, Cell Cycle, Cardiovascular System Development and Function” network were clustered to “cell death” (genes = 17/22, ES = 11.63, *p* = 9.597×10^−11^), “inflammatory response” (genes = 13/22, ES = 10.59, *p* = 6.526×10^−16^), “cell cycle arrest” (genes = 11/22, ES = 20.44, *p* = 3.759×10^−17^), and “reactive oxygen species” (genes = 11/22, ES = 10.31, *p* = 8.665×10^−11^) (Figure 2A). A majority of the genes from both networks were involved in cell death and inflammatory responses, suggesting that S-HCV drives an inflammatory response and is associated with cell death, rather than survival.

To further study the pathways driving S-HCV towards cell death, we identified the genes in the functional cluster of cell death (*n* = 17) and the inflammatory response (*n* = 13) gene by KEGG enrichment (cut-off of false discovery rate (FDR) *p* < 0.05) revealed that genes activated by S-HCV were implicated in the mitogen-activated protein kinase (MAPK) signaling pathway (genes = 6/30, *p* = 3.2 × 10^−5^), virus infection pathway (genes = 5/30, *p* = 5.3 × 10^−5^), NF-κB signaling pathway (genes = 4/30, *p* = 5.0 × 10^−5^), P53 signaling pathway (genes = 3/30, *p*= 5.7 × 10^−4^), and cancer pathway (genes = 3/30, *p* = 7.7 × 10^−4^) (Figure 2B). The six genes in the MAPK signalling pathway (highest gene count) GADD45A (Growth Arrest and DNA Damage Inducible 45 Alpha), DDIT3 (DNA Damage Inducible Transcript 3), HSPB1 (Heat Shock Protein Beta-1), AREG (Amphiregulin), NFKB2 (Nuclear Factor Kappa B subunit 2), and RELB (v-rel reticuloendotheliosis viral oncogene homolog B) were significantly upregulated in S-HCV compared to L-HCV (* *p* < 0.05) (Figure 2C). MAPK signaling genes were functionally clustered into cell cycle arrest and apoptosis. The results further confirm that S-HCV is driven towards inflammation, cell death, cell cycle arrest, and apoptosis through the upregulation of MAPK signaling genes.

### 3.3. L-HCV Promotes Migration and Metastasis via the Chemokine Signaling Pathway

**Figure 3:**
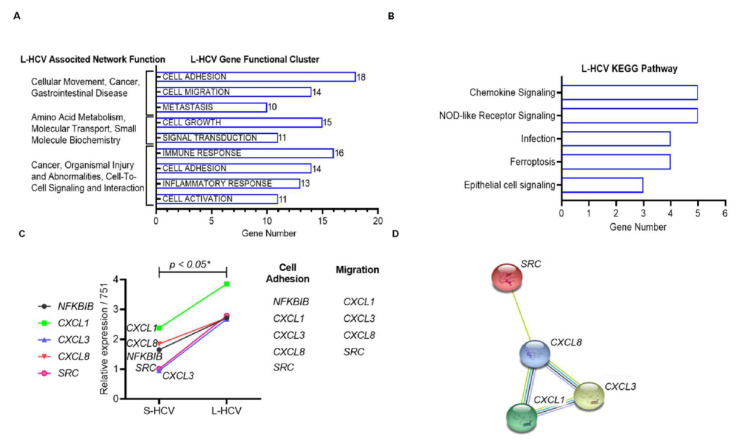
Long-term HCV (L-HCV)-associated network function gene analysis. (**A**) The top L-HCV-associated network functions and GenClip gene functional clusters (*p* < 0.05). (**B**) ShinyGO KEGG results of the top 5 enriched pathways (*p*-value cutoff (FDR) = 0.05) ranked by the number of genes from highest to lowest. (**C**) Expression of five genes (*CXCL1*, *CXCL8*, *NFKBIB*, *CXCL8*, *SRC*) in the chemokine signaling pathway compared between short-term HCV (S-HCV) and long-term HCV (L-HCV), statistical significance * *p* < 0.05. Chemokine signaling genes grouped by functional clusters. (**D**) STRING PPI network (PPI enrichment *p*-value = 0.0102) of chemokine-signaling-pathway-associated genes.

To determine the mechanisms by which L-HCV was directed towards cellular movement and cell-to-cell signalling, we identified the top enriched gene functional clusters of L-HCV-associated functional network genes, as shown in Table 2. The significantly enriched clusters (enrichment scores (ESs) with *p* < 0.05) were ranked by the highest number of genes per associated network function. Twenty-six genes associated with “Cellular Movement, Cancer, Gastrointestinal Disease” networks were clustered to “cell adhesion” (genes = 18/26, ES = 19.02, *p* = 6.917 × 10^−18^), “cell migration” (genes = 14/26, ES = 11.98, *p* = 2.078 × 10^−11^) and “metastasis” (genes = 10/26, ES = 12.03, *p* = 4.249 × 10^−9^) (Figure 3A). Twenty-two genes associated with the “Amino Acid Metabolism, Molecular Transport, Small Molecule Biochemistry” network were clustered to “cell growth” (genes = 15/22, ES = 6.81, *p* = 7.325 × 10^−8^) and “signal transduction” (genes = 11/22, ES = 4.64, *p* = 0.0006) (Figure 3A). Twenty-one genes associated with the “Cancer, Organismal Injury and Abnormalities, Cell-To- Cell Signaling and Interaction” network were clustered to “immune response” (genes = 16/21, ES = 17.72, *p* = 4.754 × 10^−13^), “cell adhesion” (genes = 14/21, ES = 14.50, *p* = 1.169 × 10^−12^), “inflammatory response” (genes = 13/21, ES = 14.16, *p* = 7.69 × 10^−17^), and “cell activation” (genes = 11/21, ES = 14.87, *p* = 1.053 × 10^−16^) (Figure 3A). These results indicate that L-HCV was implicated in cell adhesion and the immune response.

To further study the effects of these functional clusters in L-HCV, we identified the gene pathways in the functional clusters of “cell adhesion” (genes = 18), “immune response” (genes = 16) and “cell growth” (genes = 15). KEGG enrichment (cut-off of FDR *p* < 0.05) results revealed those genes to be implicated in the “chemokine signaling pathway” (genes = 5/49, *p* = 4.6 × 10^−4^), “NOD-like receptor signaling pathway” (genes = 5/49, *p* = 3.9 × 10^−4^), “ferroptosis” (genes = 4/49, *p* = 7.1 × 10^−5^), “infection” (genes = 4/49, *p* = 3.9 × 10^−4^), and “epithelial cell signaling” (genes = 3/49, *p* = 2.0 × 10^−3^) (Figure 3B). The genes *CXCL1* (C-X-C motif chemokine ligand 1), *CXCL3* (C-X-C motif chemokine ligand 3), *CXCL8* (C-X-C motif chemokine ligand 8) also known as Interleukin-8 (IL-8), *NFKBIB* (nuclear factor Kappa-B inhibitor beta), and *SRC* (proto-oncogene tyrosine-protein kinase Src) in the chemokine signaling pathway (highest gene count) were significantly upregulated in L-HCV compared to S-HCV (* *p* < 0.05) (Figure 3C). Chemokine signaling genes were functionally clustered into cell adhesion and migration. The Search Tool for the Retrieval of Interacting Genes (STRING) analysis demonstrated that L-HCV genes, through chemokine signaling, as well as *CXCL8*, significantly regulated *SRC* (protein-protein interaction (PPI) enrichment, *p*-value: 0.05 *) (Figure 3D). The results indicated that L-HCV promotes migration by altering cell adhesion through direct interactions and the upregulation of chemokine signaling genes.

### 3.4. Persistent HCV Infection Induced CXCL8 Expression and Activation of Oncogene SRC in Liver Hepatocellular Carcinoma (LIHC)

**Figure 4:**
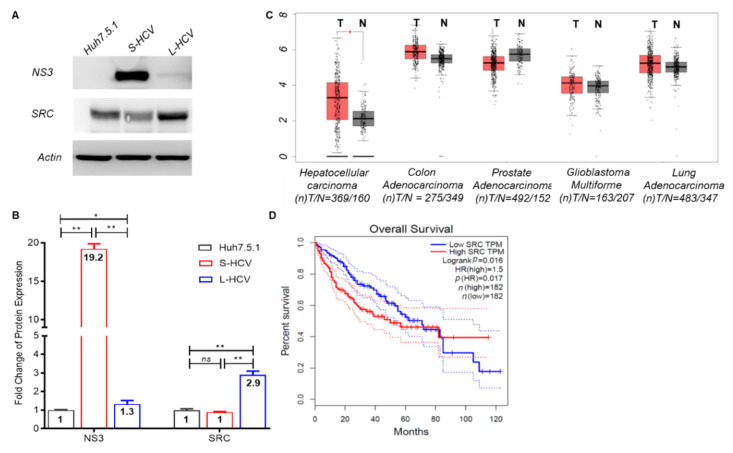
Clinical data associated with *SRC*, derived from Gene Expression Profiling Interactive Analysis (GEPIA). (**A**) Protein expression of NS3 and SRC in Huh7.5.1 cells, short-term HCV (S-HCV), and long-term HCV (L-HCV). Actin was used as a protein expression control. (**B**) Fold change in protein expression, statistical significance *p* < 0.05 *, *p* < 0.01 ** while values *p* > 0.05 were deemed non-significant (ns). (**C**) Clinical data demonstrating the regulation of SRC in liver hepatocellular carcinoma (LIHC), colon adenocarcinoma (COAD), prostate adenocarcinoma (PRAD), glioblastoma multiforme (GBM), and lung adenocarcinoma (LUAD). Red box indicates tumor (T) samples and gray box indicates non-tumor (N) samples. (**D**) Survival plot based on Kaplan–Meier analysis of SRC expression with survival outcomes. Transcripts Per Million (TPM) is a normalization method for RNA-seq. The logrank test is used for the hypothesis test in GEPIA. The hazard ratio (HR) is calculated based on Cox PH model, with the dotted lines representing the 95% confidence interval. The total number of samples is represented by *n*. Statistical significance *p* < 0.05 *.

The validation of SRC demonstrated significant overexpression in L-HCV compared to S-HCV and uninfected Huh7.5.1 cells (*p* < 0.01 **), with no significant difference between S-HCV and uninfected Huh7.5.1 cells (*p* = 0.70) (Figure 4A,B). The HCV NS3 protein was significantly upregulated in S-HCV compared to L-HCV (*p* < 0.01) and uninfected-Huh7.5.1 cells (*p* < 0.01 **). The HCV-core mRNA cycle threshold values (Ct) were 6.1 and 26.3 in S-HCV and L-HCV, respectively (data not shown). Furthermore, NS3 was significantly upregulated in L-HCV compared to uninfected Huh7.5.1 cells (*p* < 0.02 *). The cancer genome atlas (TCGA) via the GEPIA database showed that *SRC* expression was significantly upregulated in the tumor part of LIHC (T/N = 369/160) compared to COAD (colon adenocarcinoma, T/N = 275/349), PRAD (prostate adenocarcinoma, T/N = 492/152), GBM (glioblastoma multiforme, T/N = 163/207), and LUAD (lung adenocarcinoma, T/N = 483/347) (Figure 4C). Kaplan–Meier analysis demonstrated a poor survival rate in patients with HCC with high expression of SRC (log-rank, *p* = 0.016 *) (Figure 4D). These results together indicate that persistent HCV infection upregulates oncogene *SRC* in HCC patients.

**Figure 5:**
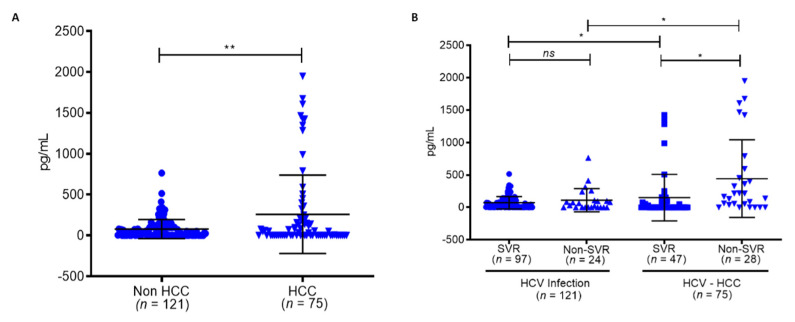
Kaohsiung Medical University Chung-Ho Memorial Hospital (KMUH) hepatitis C virus (HCV)-hepatocellular carcinoma (HCC) cohort (*n* = 196). (**A**) *CXCL8* level in non-HCC and HCC patients. (**B**) *CXCL8* level in non-HCC patients with SVR and non-SVR compared to non-HCC patients with SVR and non-SVR. Statistical significance *p* < 0.05 *, *p* < 0.001 **, while values *p* > 0.05 were deemed non-significant (ns).

To further understand the effects of persistent HCV infection on *CXCL8*, which regulates SRC in HCC patients, we collected 196 serum samples from HCV patients undergoing IFN/RBV treatment from Kaohsiung Medical University Chung-Ho Memorial Hospital. The analysis of their baseline characteristics revealed significant risk factors associated with 121 cases of HCV infection and 75 cases of HCV-HCC, which included age (*p* = 0.0001), AFP (*p* = 0.0001), cirrhosis (*p* = 0.002), and platelet number (*p* < 0.001) (Table 3). Serum levels of *CXCL8* in HCV-HCC patients were significantly elevated compared to HCV-infected patients (*p* < 0.001 **) (Figure 5A). Subgroups in the cohort, denoted as SVR and non-SVR patients with HCV treatment, represented groups with HCV elimination and prolonged HCV infection, respectively. *CXCL8* levels were significantly increased in the HCV-HCC group in SVR and non-SVR patients compared to HCV-infection SVR and non-SVR patients (*p* = 0.04 *, *p* = 0.012 *), respectively (Figure 5B). Furthermore, *CXCL8* was upregulated in non-SVR patients compared to SVR patients within the HCV-HCC group, further demonstrating that HCV persistence upregulates *CXCL8*. These findings indeed demonstrated that high levels of *CXCL8* in the HCV-HCC non-SVR group were linked with prolonged HCV infection in this cohort. Therefore, this provided an insight on the mechanism associated with HCV-HCC, namely, that it is mediated by *CXCL8* to regulate the oncogene *SRC*, promoting a cancer-favoring environment in the progression of HCC.

**Figure 6:**
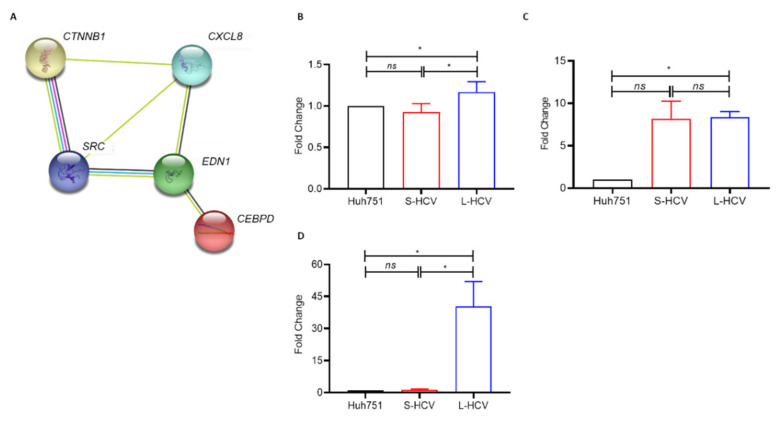
The Search Tool for the Retrieval of Interacting Genes (STRING) protein–protein interaction (PPI) network of our candidate genes, *SRC* and *CXCL8*, their interaction with cancer signatures, and their expression. (**A**) PPI enrichment of CXCL8 and SRC, and their interaction with signature genes *EDN1*, *CEBPD*, and *CTNNB1* (*p* = 6.43 × 10^−5^). Q-PCR validation of cancer signature genes’ expression of (**B**) *CTNNB1*, (**C**) *EDN1*, and (**D**) *CEBPD*. Fold changes of mRNA levels (compared to mRNA levels of Huh7.5.1 control cells) are shown as ± SD from two-to-three independent experiments (*p* < 0.05 * determined by paired *t*-tests) while values *p* > 0.05 were deemed non-significant (ns).

The TCGA results, analyzed through GEPIA, along with the KMUH clinical cohort’s *CXCL8* expression, confirm a strong positive regulatory interaction between HCV-induced chemokines promoting the upregulation of *CXCL8*, which has a downstream effect, upregulating the expression of the *SRC* oncogene. We found that prolonged HCV promotes *CXCL8* expression, which triggers and stimulates SRC expression, thereby promoting HCV-HCC. Furthermore, the PPI network illustrated that the genes *CTNNB1 (β-catenin), CEBPD (CCAAT enhancer binding protein delta), and EDN1 (endothelin 1)* were involved in *CXCL8* and *SRC* interactions, promoting HCC progression (*p* = 0.0394) (Figure 6A). Furthermore, the gene expression validation of *CTNNB1*, *EDN1*, and *CEBPD* via q-PCR showed that *CTNNB1* and *CEBPD* were significantly upregulated in L-HCV compared to S-HCV (*p* < 0.05 *) (Figure 6B–D). The results confirm that the L-HCV genes *CXCL8* and *SRC* are positively linked with cancer-promoting genes. Therefore, these findings altogether suggest that persistent HCV induces the expression of the pro-inflammatory chemokine *CXCL8*, which creates a cancer-promoting environment that triggers HCC occurrence. Thus, *CXCL8* may be a potential monitoring and prognostic marker in HCV-related HCC, and in the assessment of patients at increased risk of HCC development regulated by HCV and cancer-promoting genes.

## 4. Discussion

Hepatitis C virus replication in the liver is reflected by the amount of virus in the serum, which represents the amount of virus in the liver [25]. Direct-acting antiviral (DAA) therapy can effectively achieve SVR in HCV treatment, with a residual risk of HCC reported in patients with advanced liver disease and liver-disease related complications [26]. Various clinical and research studies have illustrated that HCV is capable of inducing HCC directly due to the functioning of proteins, or indirectly through the activation of chronic liver inflammation, which has assisted in the identification of candidate HCC targets [27]. Another clinical study has demonstrated how the eradication of HCV with IFN-based treatment leads to the impairment of hepatocarcinogenesis; however, HCC may occur even after virus eradication [28]. Whether in cohorts exhibiting non-SVR with IFN/RBV treatment, or in untreated cohorts, the indication of HCV persistence in the liver tissue is still the major risk factor for the advancement of liver fibrosis/cirrhosis to HCC. In this study, we used the JFH1 infectious cell model to illustrate the effect of the intracellular viral load and demonstrated that S-HVL drives cells towards cell death via MAPK signaling, resulting in cell cycle arrest and apoptosis. L-LVL promotes migration and cancer through chemokine signaling. This suggests that HCV viral loads have different functional mechanisms by which they alter the cellular microenvironment. Our findings are fundamental in that they suggest a direct impact on the debated issues concerning the role of direct cytopathic effects of HCV versus immune-mediated injury in HCV related pathogenesis causing liver damage.

Viruses often alter host cell mitogenic signaling to enhance the surveillance of infected cells and to establish chronic infections. Our data demonstrated that S-HCV was associated with cell death via the MAPK pathway, which regulates cell G2/M phase arrest, thereby promoting programmed cell death in apoptosis. Apoptosis and cell death are key mechanisms for the diffusion of HCV viral infections. The MAPK signaling pathway promotes mitochondrial-mediated apoptosis [29], which further supports our findings in regard to the S-HCV population promoting apoptosis. Furthermore, the maximum titer of HCV RNA has been demonstrated to reduce the number of living HCV-infected cells compared to uninfected cells. It has been demonstrated that between day 8 to 14, most of the infected cells underwent apoptotic death, which was associated with a high intracellular viral RNA titer [15]. This may highlight a causative link between the rate of HCV replication and cell death.

Lower HCV titers may worsen the outcome of HCV infection because the persistence of immune-mediated hepatic inflammation responses drives cancer development and progression [30,31]. Indeed, activated inflammatory cells promote pro-carcinogenic microenvironmental changes by releasing oxidation molecules [32]. The genes (*CXCL1*, *CXCL3*, *CXCL8*, *NFKBIB*, and *SRC*) associated with chemokine pathway signaling result in changes in the cellular microenvironment that favor cancer and transformation in L-LVL cells. *CXCL8* signaling triggers the activation of protein kinase SRC expression, promoting focus formation, anchorage-independent growth, proliferation, motility, and invasion [33]. In addition, *CXCL8* promotes actin and β-tubulin re-localization, which promotes cell spreading and motility, which are directly associated with CXCL8-induced migratory effects [34]. Patients with non-SVR and lower HCV viral loads after long-term IFN/RBV treatment demonstrate *CXCL8* expression in HCV-related HCC progression. Other studies have indicated that high *CXCL8* expression in HCC tissues was significantly associated with tumor size, differentiation, metastasis, poor overall survival, disease-free survival, and an increased risk of recurrence and mortality [35,36]. Patients with low *CXCL8* demonstrated less intrahepatic invasion and distant metastasis, lower recurrence rates, and increased overall survival time.

The GEPIA data showed the overexpression of *SRC* in hepatocellular carcinoma of the tumor variety and was significantly associated with decreased disease-specific survival. The clinical cohort demonstrated a significant change in the *CXCL8* level in patients with HCV-related HCC, with no significant change in HCV patients. These findings suggest that *SRC* and *CXCL8* levels may be potentially useful factors for monitoring the disease progression of HCV-HCC. *EDN1*, *CEBPD*, and *CTNNB1* are key players in triggering cancer-promoting pathways, including EMT and Wnt/β-catenin signaling pathways. The overexpression of *EDN1* triggers HCC and promotes angiogenesis, cell proliferation, survival, and migration [37,38]. *CEBPD* expression is induced by HCV infection; however, it does not revert to its original expression after DAA treatment [39]. HCV-induced *CEBPD* expression confers a survival advantage to cancer cells encountering ER stress [40,41]. β-catenin overexpression promotes cell proliferation, migration, and invasion and triggers apoptosis [42]. The Wnt/β-catenin pathway regulates polarity, survival, and stem cell differentiation in embryonic and adult tissue homeostasis [43]. Our findings illustrate that long-term viral load-associated genes interact with each other via intermediate genes and EMT markers.

In cellular microenvironments, HCV uses different mechanisms regulate both innate and adaptive immunity. This process involves the recruitment of antiviral immune cells in the liver, which is mainly regulated by the release of specific chemokines [44]. The regulation of their expression may be an efficient viral invasiveness mechanism to impede specific immune cell migration during viral infection [44]. CXC chemokines are upregulated in both the liver and peripheral blood during chronic HCV infection to direct migration to specific anatomical sites [45]. HCV-regulated modifications in hepatic immune cell chemotaxis in chronic-phase infection significantly alter antiviral immunity, cause liver damage, and ultimately influence the survival of both the host and the virus [44,45]. Various previous studies have shown that elevated *IL-8* is linked to the regulation of liver infection, particularly in non-SVR patients [46,47]. HCV plays a pivotal role in inducing *IL-8* gene expression in certain cellular microenvironments, which may represent a potential strategy used by HCV infection to enhance viral replication while silencing the nonspecific intra-cellular IFN antiviral response [48,49,50]. These studies support our findings in regard to *CXCL8* levels in the HCV-HCC non-SVR subgroup.

*SRC* in liver hepatocellular carcinoma was significantly upregulated in tumors and was associated with decreased disease-specific survival. Studies have shown that HCV induces gene expression or chemokines that stimulate *CXCL8* expression, which has a direct effect on SRC [44,51]. *CXCL8* induces migratory effects through the activation of protein kinase *SRC* expression to promote focus formation, anchorage-independent growth, proliferation, motility, actin and β-tubulin re-localization, and invasion [33,34]. Furthermore, *CXCL8* is often secreted by tumor cells and acts as a pro-inflammatory chemokine in chronic HCV [45,52]. These findings demonstrate that HCV-induced chemokines (*CXCL1*, *CXCL3*) stimulate *CXCL8*, which positively regulates SRC expression. Therefore, CXCL8 and *SRC* may be potentially useful markers for monitoring HCV-HCC.

There were several limitations that could not be averted in this study. First, using NGS data, we had to choose a significant cut-off that may have excluded genes that did not reach the cut-off expression level. Second, we only evaluated the gene expression levels in this study. Even though, through STRING analysis, we evaluated the potential protein–protein interactions, the phosphorylation and activation mechanisms need to be further studied to determine the effect of HCV infection in hepatocytes. Third, there are limitations to the use of in vitro data, using only Huh7.5.1 cells for HCV infection, in that they may not fully reflect conditions in the human body. We therefore intergraded clinical serum samples from patients to demonstrate and confirm that our findings may be applicable in clinical studies, particularly in HCV-HCC. However, these limitations do not have a substantial influence on our results; they are just considerations to highlight the need for care while interpreting these results.

## 5. Conclusions

**Figure 7:**
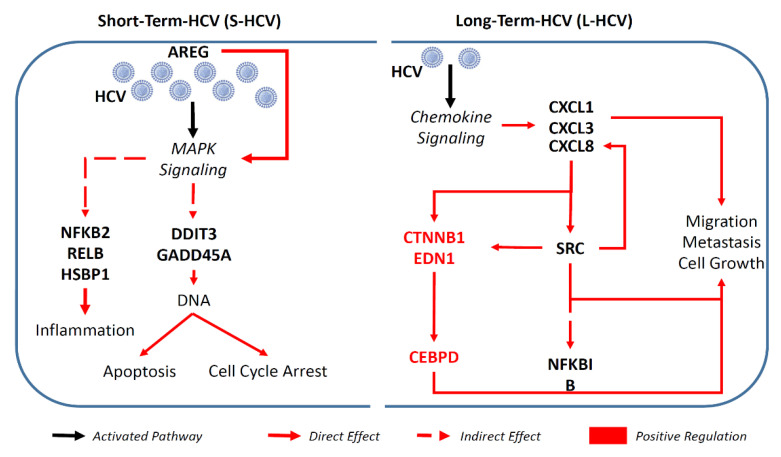
A graphical summary illustrating how intracellular HCV—short-term HCV (S-HCV), and long-term HCV (L-HCV)—activated signaling pathways with different genes, which resulted in different effects in cellular microenvironments, respectively.

A graphical summary is shown in Figure 7. Our study indicated that short-term-HCV high-viral-load infection induces the expression of genes implicated in cell death and apoptosis via MAPK signaling, and HCV persistence induces the expression of genes that alter cell adhesion and promote migration via the *CXCL8-SRC* signaling pathway. The clinical GEPIA database provides strong evidence of SRC expression in liver hepatocellular carcinoma progression and poor survival. Furthermore, the cohort study demonstrated *CXCL8* expression in HCV-HCC, especially in the non-SVR group. Therefore, this study not only provides more evidence on the role of HCV in hepatocellular carcinogenesis, but has also revealed long-term viral effects on the gene expression signature. This study can provide the application of follow-up biomarkers in non-SVR patients treated with RBV/IFN for the prevention of HCC.

## Figures and Tables

**Table 1:** Short-term high viral load (S-HVL)-associated network functions.

ID	Associated Network Functions	Score	Molecules	Gene List
**1**	Cell Morphology, Embryonic Development, Hematological System Development and Function	47	24	*AGR2*, *HDAC9*, *PPARGC1A*, *ANKRD1*, *ISG15*, *RASD1*, *AREG*, *ISG20*, *RELB*, *BBC3*, *MIR17HG*, *SESN2*, *BTG2*, *MT-ND2*, *SLC1A5*, *CREB5*, *NFKB2*, *TNFRSF12A*, *DUSP10*, *NFKBIE*, *TUBA1A*, *EPB41L4A-AS1*, *NUPR1*, *UCA1*
**2**	Cell Death and Survival, Cell Cycle, Cardiovascular System Development and Function	42	22	*ASNS*, *ERK*, *DDIT3*, *ATF3*, *GADD45A*, *DDIT4*, *BEX2*, *GDF15*, *EIF1*, *BHLHE40*, *HSPB1*, *ELF3*, *CTH*, *JUNB*, *STC2*, *CXCL2*, *KLF10*, *TNFAIP3*, *KLF6*, *MAFF*, *TRIB3*, *SH3BP2*

Score: Networks are scored based on the number of Network Eligible Molecules they contain. Focus Molecules: Genes that are part of our input list. Gene List: Focus Molecules Genes.

**Table 2:** Long-term low viral load (L-LVL)-associated network functions.

ID	Associated Network Functions	Score	Focus Molecules	Gene List
**1**	Cellular Movement, Cancer, Gastrointestinal Disease.	43	26	*AJUBA*, *H2AC18/H2AC19*, *CTNND2*, *ANXA3*, *HOXB7*, *EIF4EBP1*, *CD55*, *LASP1*, *EPCAM*, *CD9*, *LIMA1*, *GPX2*, *CDH1*, *MT2A*, *ZFAS1*, *CDH17*, *OCLN*, *TFF3*, *CDH2*, *RCN1*, *TINAGL1*, *CLDN7*, *SEMA3C*, *TRIM31*, *TSPAN8*, *VTN*
**2**	Amino Acid Metabolism, Molecular Transport, Small Molecule Biochemistry.	34	22	*APOC3*, *KLF5*, *EDN1*, *BMF*, *PARP9*, *EPS8L2*, *C9orf72*, *PDCD4*, *GSTA2*, *CCR6*, *PMAIP1*, *HLA-E*, *CEBPD*, *S100A11*, *SLC3A2*, *CHMP4C*, *S100A6*, *SLC7A11*, *CYP1A1*, *SAT1*, *SOCS2*, *SPINT1*
**3**	Cancer, Organismal Injury and Abnormalities, Cell-to-Cell Signaling and Interaction.	32	21	*ANXA1*, *IL18*, *CXCL8*, *BLVRA*, *IL32*, *F2RL1*, *CAV1*, *KYNU*, *G0S2*, *CXCL1*, *MARVELD3*, *HMOX1*, *CXCL3*, *MDK*, *HOXB9*, *NFKBIB*, *OPTN*, *TSC22D3*, *NUB1*, *SRC*, *UBD*

Score: Networks are scored based on the number of Network Eligible Molecules they contain. Focus Molecules: Genes that are part of our input list. Gene List: Focus Molecules Genes.

**Table 3:** Clinicopathological characteristics of HCV-HCC patients in a cohort treated with pegylated interferon (IFN).

Clinical Factors	HCV Infection (*n* = 121)	HCV-HCC (*n* = 75)	*p*-Value	HCV Infection (*n* = 121)			HCV-HCC (*n* = 75)		
				SVR (*n* = 97)	Non-SVR (*n* = 24)	*p*-Value	SVR (*n* = 47)	Non-SVR (*n* = 28)	*p*-Value
Age	49.29 ± 11.14	58.6 ± 8.18	0.0001 *	48.74 ± 11.20	51.50 ± 10.56	0.646	60.15 ± 7. 72	56.00 ± 8.40	0.0152 *
Sex (F/M)	58/63	29/46	0.8331	45/52	13/11	0.357	14/33	15/13	0.0149 *
AFP (IU/L)	10.99 ± 15.69	36.53 ± 96.70	0.0001 *	10.66 ± 16.49	12.31 ± 10.89	0.8975	21.09 ± 31.18	62.45 ± 151.19	0.9945
BMI (Kg/m^2^)	40.57 ± 5.55	41.61 ± 6.20	0.9461	40.59 ± 5.43	40.52 ± 5.90	0.7201	41.90 ± 7.06	41.12 ± 4.49	0.2522
Cirrhosis (N)	28/121	50/75	0.002 *	21/97	7/24	0.25	27/47	23/28	0.0829
GOT (IU/L)	94.45 ± 63.40	111.17 ± 64.93	0.9441	96.48 ± 66.42	86.21 ± 46.76	0.239	108.72 ± 71.23	115.21 ± 53.69	0.7221
GPT (IU/L)	153.71 ± 120.65	145.88 ± 95.24	0.2069	159.89 ± 125.90	128.75 ± 89. 11	0.1308	149.17 ± 109.99	140.35 ± 64.78	0.4912
GGT (IU/L)	58.72 ± 54.13	95.65 ± 72.80	0.9996	60.18 ± 58.31	52.74 ± 30.06	0.2893	83.42 ± 63.40	116.03 ± 83.74	0.5529
PLT (10^3^/µL)	178.86 ± 62.49	123.67 ± 40.47	<0.001 *	185.61 ± 61.57	151.58 ± 57.23	0.059	129.12 ± 38.06	114.50 ± 43.37	0.0919

AFP, Alpha-fetoprotein: BMI, Body mass index: Tx, Treatment: GPT, glutamate-pyruvate transaminase: GOT, glutamic oxaloacetic transaminase: DDT, gamma-glutamyl transferase: PLT, platelet. Statistical significance < 0.05 *.

## Supplementary Materials

The following are available online at https://www.mdpi.com/article/10.3390/biomedicines9101446/s1. Figure S1: Flow Chart Study Design. Human hepatoma 7.5.1 (Huh 751) were cultured and infected with JFH1-EYFP for five days and distinguished by fluorescence activated cell sorting (FACS) set to 0–20% low intensity for low viral load and 80–100% for high intensity for high viral load. Low viral load cells were continuously passaged, cultured and sorted on day 30 to establish long-term HCV (L-HCV) cells. Uninfected Huh7.5.1, S-HCV, eL-HCV and L-HCV were harvested for RNA sequencing and expression profiling. The differentially expressed genes with fold change > 2.0 and statistical significance *p* < 0.05 * were selected for enrichment analyses using different bioinformatics resources. Finally, L-HCV candidate genes were searched for in Gene Expression Profiling Interactive Analysis (GEPIA) to observe their expression in different cancers, Figure S2: Distinguishing Intracellular HCV infectious cells, Figure S3: Establishing long-term HCV (L-HCV) cells, Table S1: The primer sequences of genes for Real-time PCR, Table S2: The the Fold change (FC) ≥ −2 or ≤ 2 of NGS mRNA expression in S-HCV, eL-HCV, and L-HCV cells.
