# Supplementary material for: The Persistence of Hepatitis C Virus Infection in Hepatocytes Promotes Hepatocellular Carcinoma Progression by Pro-Inflammatory Interluekin-8 Expression
Source: Biomedicines. 2021 Oct 11;9(10):1446. doi: 10.3390/biomedicines9101446 (PMC8533125; doi:10.3390/biomedicines9101446)
Supplement: Supplementary file 1 [file biomedicines-09-01446-s001.zip › biomedicines-1385051-supplementary.pdf]

Supplementary Figures:

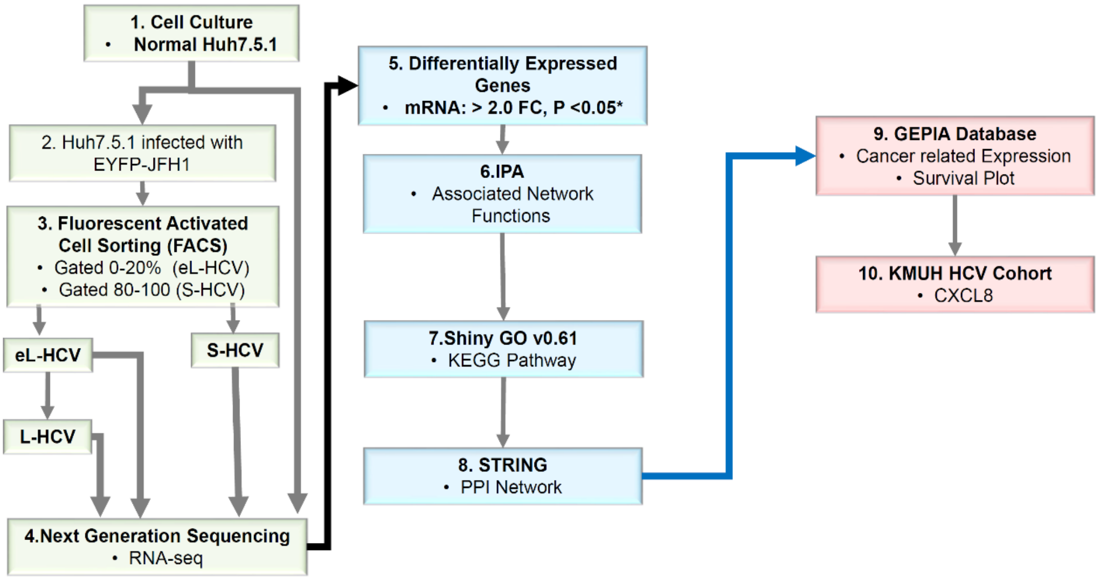

Supplementary Figure S1. Flow Chart Study Design. Human hepatoma 7.5.1 (Huh 751) were cultured and infected with JFH1-EYFP for five days and distinguished by fluorescence activated cell sorting (FACS) set to 0%-20% low intensity for low viral load and 80%-100% for high intensity for high viral load. Low viral load cells were continuously passaged, cultured and sorted on day 30 to establish long-term HCV (L-HCV) cells. Uninfected Huh7.5.1, S-HCV, eL-HCV and L-HCV were harvested for RNA sequencing and expression profiling. The differentially expressed genes with fold change > 2.0 and statistical significance p < 0.05* were selected for enrichment analyses using different bioinformatics resources. Finally, L-HCV candidate genes were searched for in Gene Expression Profiling Interactive Analysis (GEPIA) to observe their expression in different cancers.

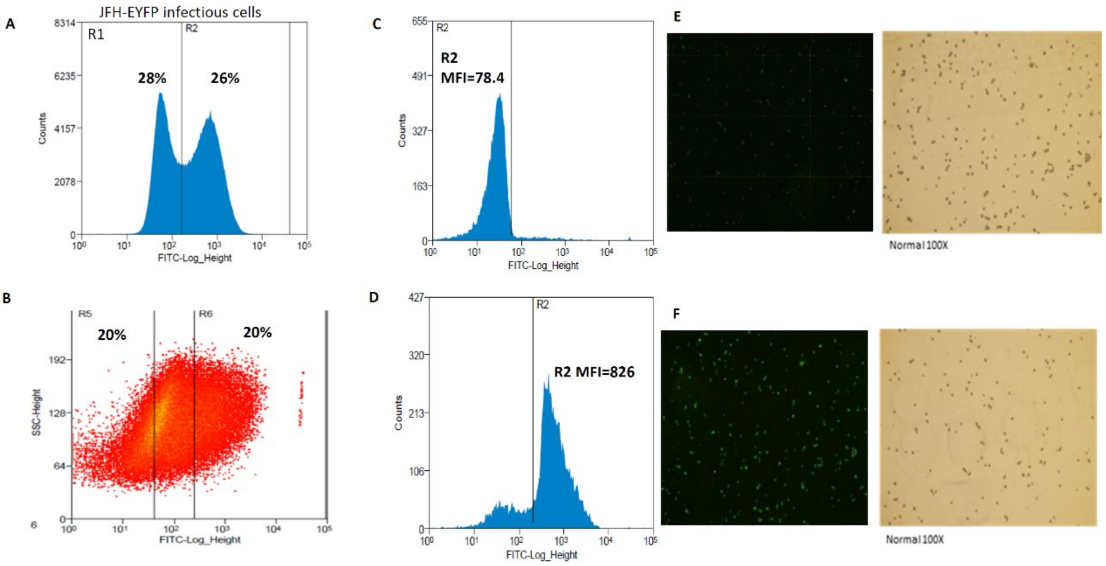

Supplementary Figure S2. Distinguishing Intracellular HCV infectious cells. (A) A histogram of total JFH-EYFP infectious cells by fluorescence activated cell sorting (FACS). **(B)** Scatter plot by FACS showing infectious population. The cells were gated at 20% (0%-20%) for low viral load and (80%-100%) for high viral load. **(C)** The mean fluorescence intensity (MFI) =78 for low viral load. Low viral load populations demonstrated very low fluorescence levels. **(D)** The mean fluorescence intensity (MFI) =82.6 for high viral load. High viral load populations demonstrated high fluorescence levels.

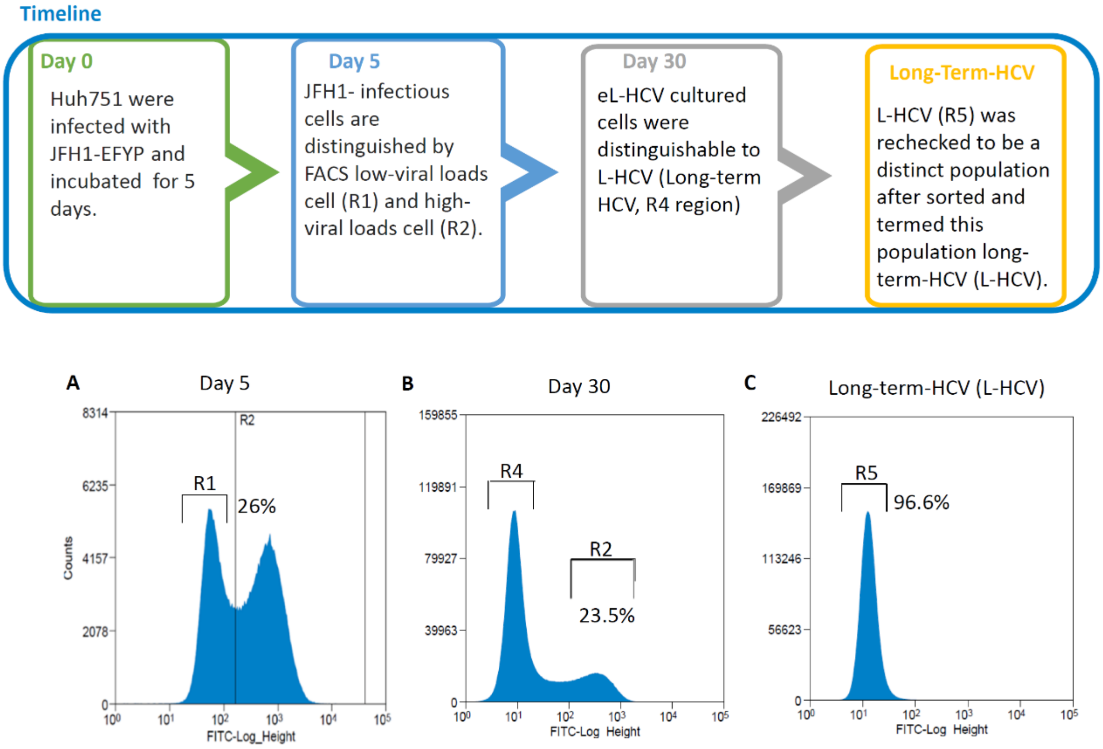

Supplementary Figure S3. Establishing long-term HCV (L-HCV) cells. (A) A histogram on day 5 of total infectious cell to obtain low viral load cells (R2 region, 26% of total infections cells) referred to as early long-term HCV (eL-HCV). **(B)** A histogram on day 30 of day 5 derived eL-HCV cells showing an increase of low viral load cells (R4 region) and a decreased of high viral load cells (R2 region, 23.5%) by FACS. **(D)** A histogram of low viral load cells (R4 region) on day 30 after sorting and confirmed to be low viral load referred to as long-term HCV (L-HCV) showing 96.6 % isolation efficiency of L-HCV cells.

Supplementary Methods:

Data analysis tools:

Ingenuity Pathway Analysis (IPA) software (Ingenuity Systems Inc., Redwood City, CA, USA) allows for the "Core Analysis" of a set of genes of interest, based on integrated literature searches that already exist and are constantly updated. GenCLiP 3 is a web server and is updated version of GenCLiP 2.0 analysis of human gene functions and regulatory networks. It provides accurate recognition of molecular interactions with polarity and directionality from PubMed database. It is available at: http://ci.smu.edu.cn/genclip3/. ShinyGO is an intuitive and graphical web application that allows to understand a gene list through integration of gene ontology (GO) enrichment analysis as KEGG. It is available at: http://bioinformatics.sdstate.edu/go/. The Search Tool for the Retrieval of Interacting Genes (STRING) database (version 11) contains analysis and combines protein-protein interactions (PPI), either direct or indirect, interactions while focusing on functional association. Gene Expression Profiling Interactive Analysis (GEPIA) is a customizable and interactive tool with functions such as differential expression analysis and patient survival analysis. GEPIA is available at: http://gepia.cancer-pku.cn/.

Supplementary Table S1. The primer sequences of genes for Real-time PCR.

Primers	Sequence
CTNNB1_F	CCCACTAATGTCCAGCGTTT
CTNNB1_R	AACGCATGATAGCGTGTCTG
CEBPD_mRNA_F	AGCGCAACAACATCGCCGTG
CEBPD_mRNA_R	GTCGGGTCTGAGGTATGGGTC
EDN1_mRNA_F	AGCTGTCCAAGTCAGACGC
EDN1_mRNA_R	GCCCAAGTGCCCTTTTAACG
GAPDH_F	GAAGGTGAAGGTCGGAGT
GAPDH_R	GAAGATGGTGATGGGATTTC

Supplementary Table S2. The the Fold change (FC) ≥ −2 or ≤ 2 of NGS mRNA expression in S-HCV, eL-HCV, and L-HCV cells.

Groups	FC ≥ −2	FC≥ 2	
S-HCV	LINC00504	ELF3	DDIT4
	MIR17HG	OXA1L	SHC2
	SPP2	HSPB1	BTG2
	MT-ND2	PPARGC1A	UBALD2
	UGT8	HS1BP3	JUNB
	AC092669.3	CTB-147N14.6	SCARNA9
	AL109927.1	BHLHE40	KIF5C
	TBC1D8B	MAFF	UAP1L1
	NBPF9	PATL1	RELB
	ATP9B	SULT1C4	CREB5
	RP11-703G6.1	MRAS	NFKB2
	CYP3A5	DUSP10	KLF6
	MT-ND1	NFKBIE	TRIB3
	WDPCP	RNASEK	GTPBP2
		UCA1	PTPRH
		BBC3	RASD1
			RP11-108M9.4
eL-HCV	PVT1	PTMS	SERPIND1
	FOSB	RP11-187E13.1	CH17-270A2.2
	CYSLTR1	FAM134B	RP11-692C24.2
	ARG1	SLC38A3	IGF2
		KIAA0195	
L-HCV	HIST1H2BM	AFP	OPTN
	HIST1H2AE	RCN1	JDP2
	HIST1H2BB	HSBP1L1	VTN
	HIST1H1A	SPINK1	PARP9
	HIST1H2AH	KRT23	CAV1
	HIST3H2A	C1orf35	TMEM69
	HIST1H3A	NUB1	ADPRHL1
	HIST1H2AD	SNAP29	SKAP2
	HIST1H2AM	CRTAP	RFPL4B
	HIST1H1B	PDCD4	C6orf52
	HIST1H2AB	SLC3A2	CDH2
	RP11-148B6.1	CTNND2	RP11-59D5__B.2
	RP11-468E2.2	SMAGP	CTC-246B18.10
	HIST1H2BO	LASP1	RP9
	APOA1-AS	RWDD3	KCNQ2
	RP11-1151B14.3	C9orf72	SEMA3C
	IFT122	VPS13C	SPATA20
	HIST2H3D	EIF4EBP1	CD9
	SARDH	IMMP1L	SPSB1
	HIST1H2BJ	MED10	ABCA7
	HIST2H2BE	DHRS3	MOSPD1
	MVD	GABARAP	PPP1R1C
	ETV1	MYO1D	TNFAIP2
	HIST1H2AK	WDR53	BLVRA
	CCR6	NSUN5	PMAIP1
	ADAMTS12	LRRC42	THG1L
	HIST2H2AA4	ZNF559-ZNF177	APOC3
		MYO5B	MT1E

Groups	FC ≥ −2	FC≥ 2	
L-HCV		HMOX1	MPP1
		UBD	ESPN
		ZNF528	EPCAM
		FZD6	GPX2
		TFF3	SPX
		FBN1	AJUBA
		CAPN2	TMEM154
		AKR1D1	TRAPPC2B
		MARVELD3	TINAGL1
		SLC44A3	C4BPB
		HLA-E	SRC
		BMF	ANXA1
		ELOVL7	FAM47E-STBD1
		GJD3	STX1A
		STX3	UPF3B
		MDK	ICA1
		CLRN3	RP11-689K5.3
		CHMP4C	FSTL1
		GUCY2C	FZD5
		SAT1	CYP1A1
		GPHN	AC084809.2
		OCLN	DOCK11
		IL32	RAB37
		EDIL3	MT1F
		FAM50A	EDN1
		LIMA1	HYI
		CXCL1	PPP1R14A
		CDH17	TMEM45B
		NPNT	TSC22D3
		TRIM31	RASGEF1B
		NTN4	IL18
		F2RL1	PI3
		KLF5	S100A11
		CDH1	LGALS2
		S100A6	C2orf15
		MT2A	HOXB7
		CXCL8	LRRC31
		SPINT1	B3GNT8
		CHAC1	HEPH
		TSPAN8	CA4
		CEBPD	ANXA3
		TFF1	CXCL3
		C6orf48	GSTA2
		HPN	KLF11
		RP1-45I4.3	UNC93A
		SLC7A11	EPS8L2
		C4BPA	SLC7A7
		FAM47E	G0S2
		MAP7	C19orf33
		FMOD	IDNK

Groups	FC ≥ −2	FC≥ 2	
L-HCV		HOXB9	SOCS2
		CLDN7	TXNIP
		SLPI	ACE2
		TMEM27	KYNU
		NPPB	DBNDD2
		PHGR1	RP11-452F19.3
		NFKBIB	ZFAS1
		PXDC1	AC007405.6
		CD55	
